# Supplementary material for: Nuclear-cytoplasmic compartmentalization of the herpes simplex virus 1 infected cell transcriptome is co-ordinated by the viral endoribonuclease vhs and cofactors to facilitate the translation of late proteins
Source: PLoS Pathog. 2018 Nov 26;14(11):e1007331. doi: 10.1371/journal.ppat.1007331 (PMC6283614; doi:10.1371/journal.ppat.1007331)
Supplement: S7 Table — (DOCX) [file ppat.1007331.s007.docx]

| **Gene** | **Forward** | **Reverse** |
| --- | --- | --- |
| VP22 | cgcgatgagtacgaggatct | gagggcataatccgactcgt |
| ICP27 | gatcgactacgcgacccttg | gcagacacgactcgaacact |
| ICP22 | tgtgcaagcttccttgtttg | ggcatcggagatttcatcat |
| TK | tacccgagccgatgacttac | gttatctgggcgcttgtcat |
| vhs | atccaacacaatatcacagcccatcaacag | cgccaacctctatcacaccaacacg |
| UL47 | gcatccgccaaaaagctcat | ggtatatcacgggcgatggg |
| gB | gtctgcaccatgaccaagtg | ggtgaaggtggtggatatg |
| gC | gggtccgtcccccccaat | cgttaggttgggggcgct |
| gD | tacaacctgaccatcgcttg | gcccccagagacttgttgta |
| gE | ccacgcacatggagacttc | gacgaggatgacaatgacg |
| RPLP0 | actctgcattctcgcttcct | ggactcgtttgtacccgttg |
| GAPDH | acagtcagccgcatcttc | ctccgaccttcaccttcc |
| β actin | ctggaccggtcaaggtgaca | agggacttcctgtaacaatgca |
| 18s | ccagtaagtgcgggtcataagc | gcctcactaaaccatccaatcgg |
| luciferase | cagccagatgcaatcaatgcc | tggaatcctgaacccacttct |
| IFIT1 | cctgaaaggccagaatgagg | tccaccttgtccaggtaagt |
| MMP1 | HP100549 (SinoBiological) |  |
| MMP3 | HP100493 (SinoBiological) |  |
| COL6A2 | HP205628 (OriGene) |  |
| Herc5 | PPH08668F (Qiagen) |  |
| IFIT2 | PPH02853A (Qiagen) |  |
| CHPF2 | PPH10342B (Qiagen) |  |
| GPR39 | HP205373 (OriGene) |  |

**S7 Table**. Primer pairs used for qRT-PCR
