# Supplementary figures and images for: Nuclear-cytoplasmic compartmentalization of the herpes simplex virus 1 infected cell transcriptome is co-ordinated by the viral endoribonuclease vhs and cofactors to facilitate the translation of late proteins
Source: PLoS Pathog. 2018 Nov 26;14(11):e1007331. doi: 10.1371/journal.ppat.1007331 (PMC6283614; doi:10.1371/journal.ppat.1007331)

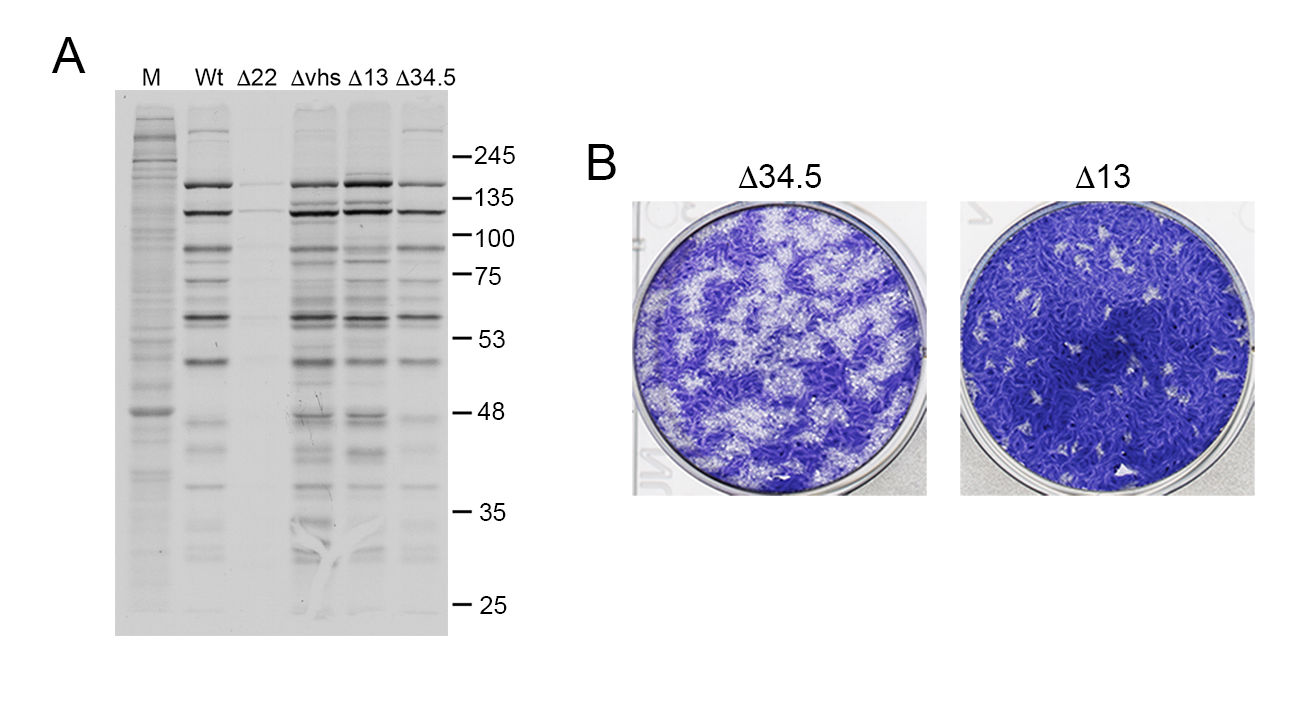

Supplement: S1 Fig — Translational shutoff (A) and plaque size phenotype (B) of HSV1 lacking either the UL13 or ICP34.5 gene on HFFF cells. (TIF) [file ppat.1007331.s008.tif]

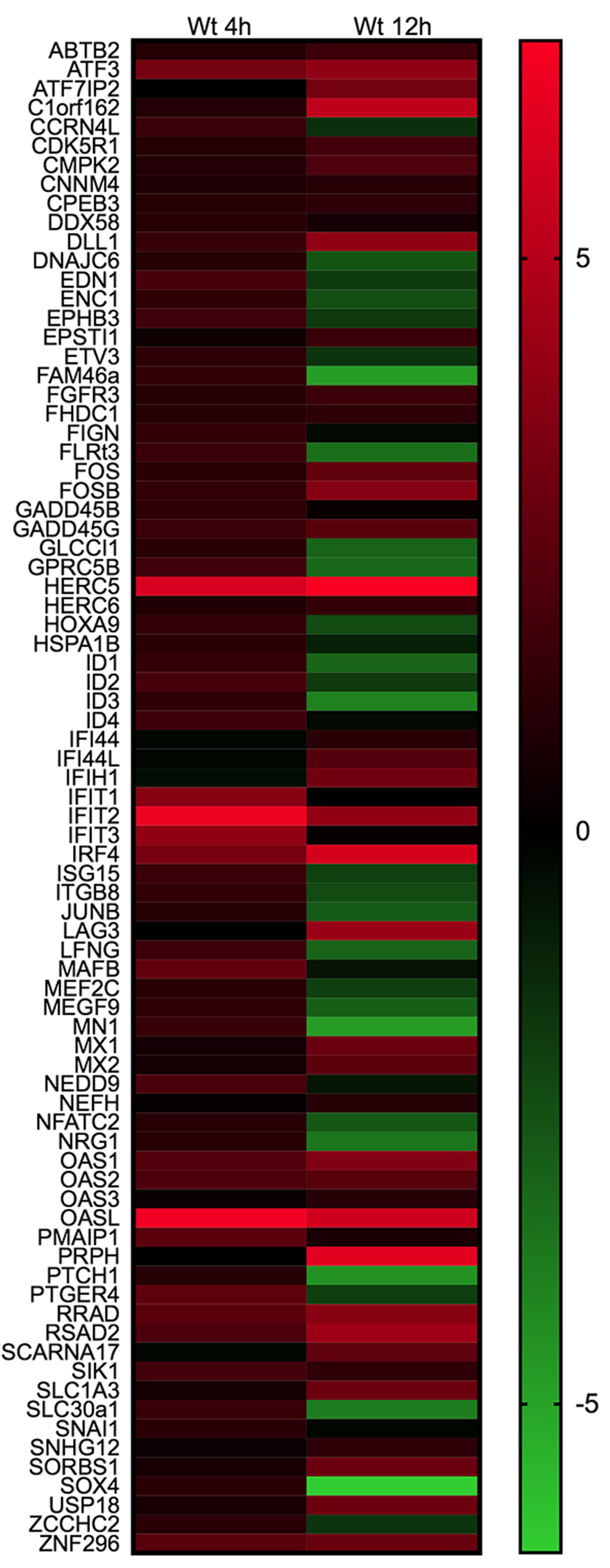

Supplement: S2 Fig — (TIF) [file ppat.1007331.s009.tif]

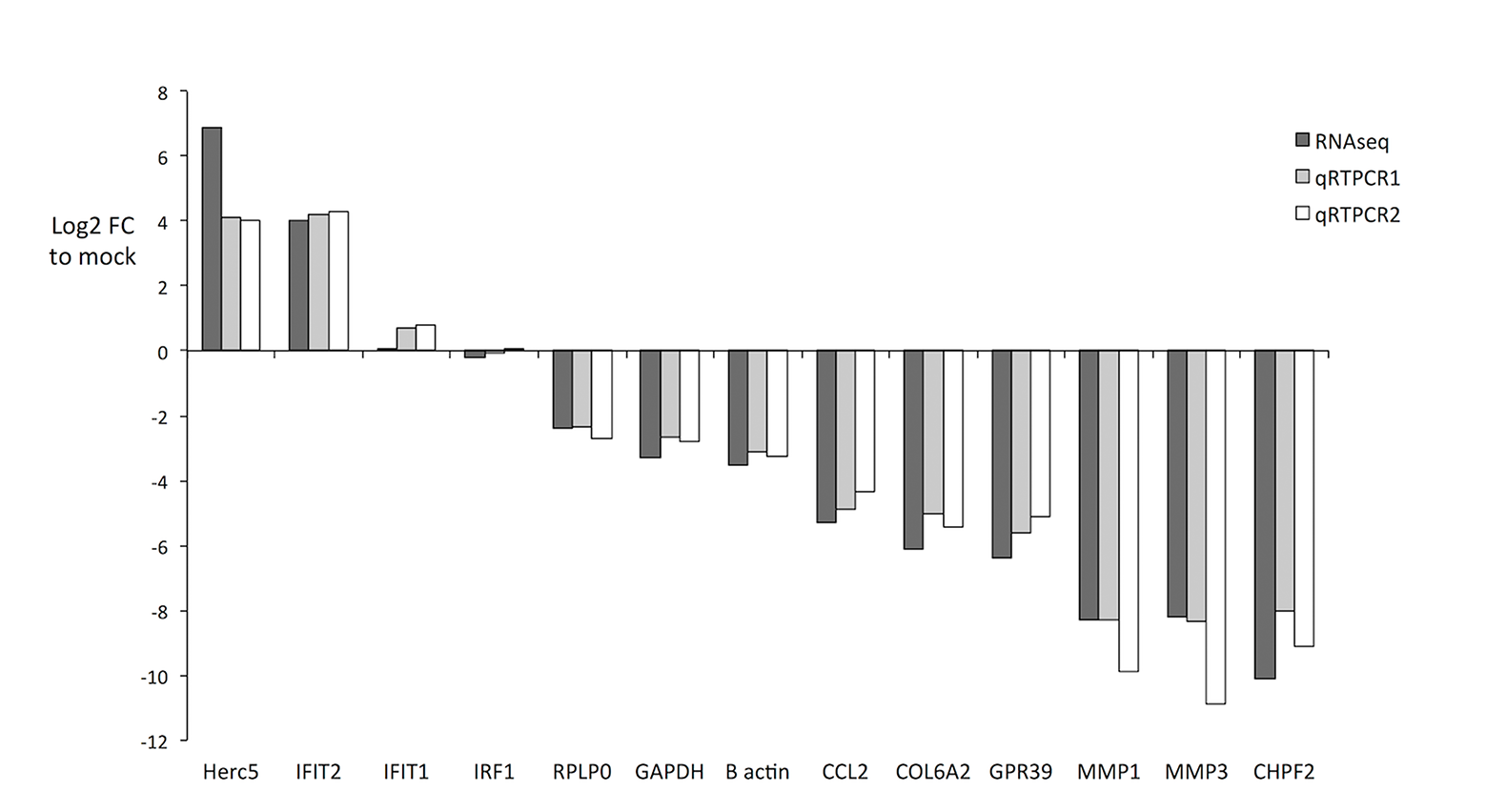

Supplement: S3 Fig — Two replicate RNA samples were subjected to qRT-PCR using primers for the indicated transcripts, and the Log2 FC compared to that determined in the RNAseq experiment detailed in S2 Table. (TIF) [file ppat.1007331.s010.tif]

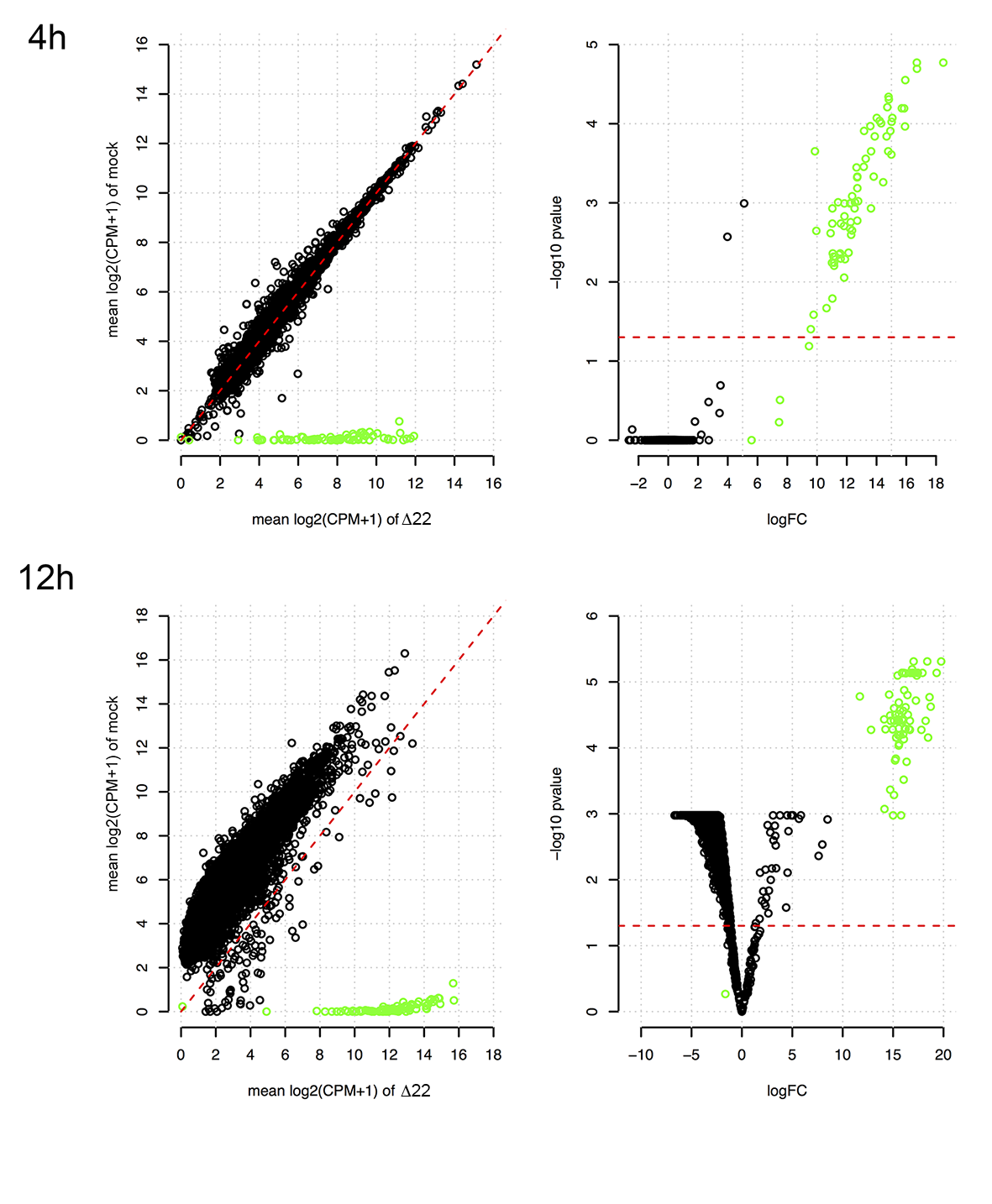

Supplement: S4 Fig — Differential expression analysis of cell and virus transcripts was conducted using EdgeR as described in Methods. Differences in the number of reads mapped to cell (black circles) and virus (green circles) transcripts were plotted as scatter plots (left hand panel) and volcano plots (right hand panel) comparing results at 4 and 12 hours to uninfected cells. (TIF) [file ppat.1007331.s011.tif]

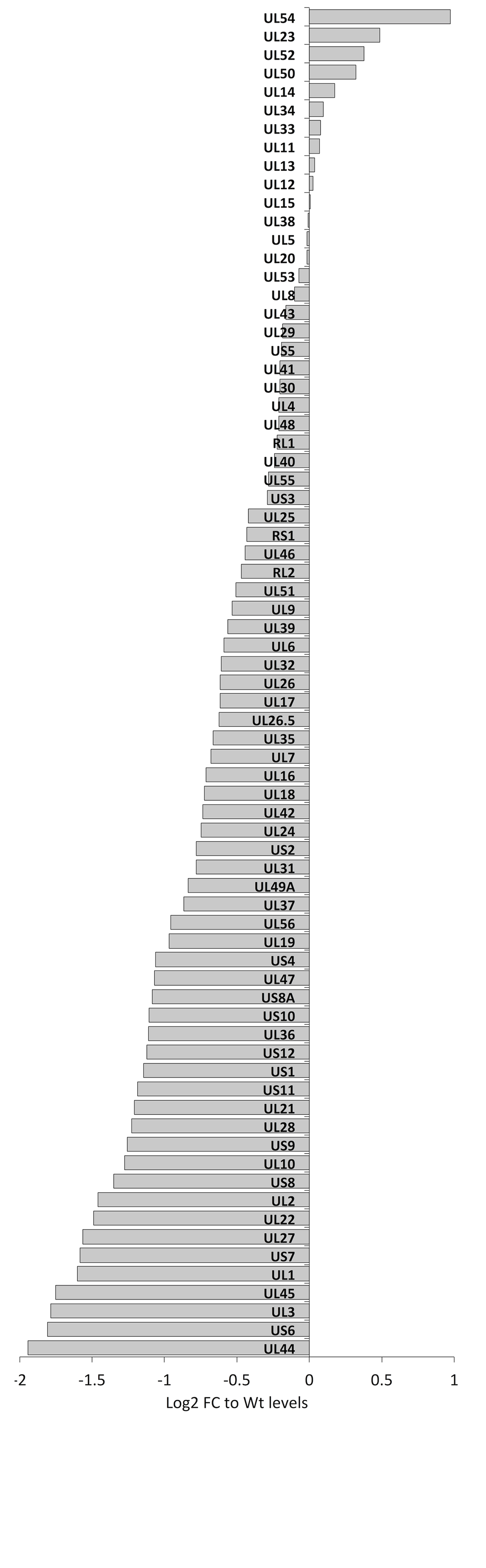

Supplement: S5 Fig — (TIF) [file ppat.1007331.s012.tif]
